# Priming for Performance: Valence of Emotional Primes Interact with Dissociable Prototype Learning Systems

**DOI:** 10.1371/journal.pone.0060748

**Published:** 2013-04-30

**Authors:** Marissa A. Gorlick, W. Todd Maddox

**Affiliations:** 1 Psychology Department, University of Texas, Austin, Texas, United States of America; 2 Institute for Neuroscience, University of Texas, Austin, Texas, United States of America; 3 Center for Perceptual Systems, University of Texas, Austin, Texas, United States of America; 4 Institute for Mental Health Research, University of Texas, Austin, Texas, United States of America; University of California, Davis, United States of America

## Abstract

Arousal Biased Competition theory suggests that arousal enhances competitive attentional processes, but makes no strong claims about valence effects. Research suggests that the scope of enhanced attention depends on valence with negative arousal narrowing and positive arousal broadening attention. Attentional scope likely affects declarative-memory-mediated and perceptual-representation-mediated learning systems differently, with declarative-memory-mediated learning depending on narrow attention to develop targeted verbalizable rules, and perceptual-representation-mediated learning depending on broad attention to develop a perceptual representation. We hypothesize that negative arousal accentuates declarative-memory-mediated learning and attenuates perceptual-representation-mediated learning, while positive arousal reverses this pattern. Prototype learning provides an ideal test bed as dissociable declarative-memory and perceptual-representation systems mediate two-prototype (AB) and one-prototype (AN) prototype learning, respectively, and computational models are available that provide powerful insights on cognitive processing. As predicted, we found that negative arousal narrows attentional focus facilitating AB learning and impairing AN learning, while positive arousal broadens attentional focus facilitating AN learning and impairing AB learning.

## Introduction

Real-world learning is often performed under exhilarating or threatening conditions creating goal-irrelevant emotional arousal that likely affects behavior. Emotional arousal affects a wide array of cognitive processes [1]–[4], yet most learning research is conducted under low-arousal leaving a critical gap in our understanding. This study examines the effects of positive and negative emotional priming on learning in dissociable perceptual-representation-mediated (perception-mediated) and declarative-memory-mediated (memory-mediated) learning systems [5], [6] using accuracy and computational modeling. To anticipate, we found that positive emotional arousal enhanced perception-mediated learning, but hindered memory-mediated learning, whereas negative emotional arousal enhanced memory-mediated learning but hindered perception-mediated learning. Computational modeling suggested this was due to positive arousal broadening and negative arousal narrowing attention.

### Emotional Arousal, Perception, and Cognition

The effects of emotional arousal are inconsistent with emotional arousal sometimes accentuating and sometimes attenuating cognitive and perceptual processing [3], [7]–[10]. To consolidate these findings, Mather and colleagues proposed an *Arousal-Biased Competition* theory (ABC) [11], [12]. ABC assumes that arousal enhances ongoing competitive attention between stimuli. In general, *high priority* stimuli receive more attentional resources at the expense of *low priority* stimuli. Arousal further exaggerates this attentional polarity.

Attentional priority is not limited to the source of arousal and is determined by both bottom-up (e.g., contrast) and top-down (e.g., goal) processes. For example, arousing sounds played before high and low contrast letters led to enhanced perception of high priority high contrast letters and decreased perception of low priority low contrast letters compared to controls [11]. Another study presented participants with instructions to a) “remember the location of words” or b) “remember the order of words” presented in a list [13]. Arousing sounds played before word presentation selectively enhanced memory for goal-relevant word features compared to controls. Those asked to remember the order demonstrated better memory for the order and worse memory for the location than controls. This pattern reversed in the word location condition. Together this demonstrates that *arousal exaggerates attentional competition for both bottom-up perceptual biases and top-down memory biases*. Arousal likely influences perception-mediated and memory-mediated learning systems as well, but to date little work has focused on these effects.

### Valence

Mather and colleagues acknowledge that ABC may require modification to account for other factors such as valence [11]. Positive and negative valence has been shown to affect the *scope* of attention differently [14], however see [15]. Positive arousal broadened the scope of attention, cognition, and action while negative arousal narrowed these aspects [16]. Supporting this idea, the *Weapon Focus Effect* has robustly demonstrated that those involved in an altercation remember the weapon but struggle to identify the face of the perpetrator [8]. On the other hand, Isen and colleagues demonstrated that those in a good mood applied a broad definition to sort words into groups compared to controls (e.g., sorting the word “camel” into the group “vehicle”). Thus, while attention for high-priority items is enhanced, it is unclear whether scope of this attention is valence-dependent.

It is likely that the scope of attention will affect performance within perception-mediated and memory-mediated learning systems differently. The perception-mediated learning system depends on broad attentional scope to develop a global perceptual representation. On the other hand, memory-mediated learning depends on narrow attentional scope to target concrete feature dimensions. Thus, negative arousal's narrow attentional scope may facilitate memory-mediated learning while positive arousal's broad attentional scope may facilitate perception-mediated learning.

### Prototype Learning

Prototype learning offers a valuable paradigm to test the effects of positive and negative arousal on attention and performance within the two systems. Participants are asked to categorize exemplars that have been distorted from one or more distinct prototypes. Declarative-memory-mediated (AB) and perceptual-representation-mediated (AN) prototype learning systems have been dissociated behaviorally [17], functionally [5], and in clinical populations [18], [19]. AB learning is memory-mediated, available to conscious control, and optimal task performance requires narrow attention to develop verbalizable rules. In AB learning, category structures are trained using exemplars distorted from two distinct prototypes (A and B). AN learning is perception-mediated, is not available to conscious control, and optimal task performance relies on broad attention to develop familiarity. In AN learning, category structures are trained using exemplars distorted from only one prototype (A).

### The Present Research

The goal of this research is to investigate how task-irrelevant positively and negatively emotionally-arousing primes affect performance in perception- and memory-mediated learning systems. In addition, we use computational models to provide insights onto arousal's effects on attention. During training we presented either a positively or a negatively arousing emotional prime before each stimulus. During test participants are asked to categorize novel stimuli without primes.

Because positive emotional primes have been shown to broaden attention, we predict that attention will be evenly distributed across features. On the other hand, because negative emotional primes have been shown to narrow attention, we predict that attention will be limited to a small number of features. Importantly, these predictions apply to both AN and AB learning tasks, but are predicted to have very different effects. Specifically, in the AB task, we predict that negative emotional primes will narrow attention for features from category A and category B which is ideal for verbalizable rule development yielding high accuracy. Positive emotional primes will broaden attentional focus for category A and category B making it difficult to develop concrete rules yielding low accuracy. In the AN task, we predict that the positive emotional primes will broaden attention facilitating a global impression of category A yielding high accuracy. Negative emotional primes will narrow attention creating a weak perceptual-representation of category A yielding low accuracy. Thus, we predict an interaction between task (AB, AN) and valence of primes (positive, negative), where negative arousal enhances performance in the declarative memory-mediated AB task and positive arousal enhances performance in the perception-mediated AN task.

## Methods

### Participants

One hundred forty five undergraduates age 18–35 participated in the AB condition (*n_NegArs_* = 34, *n_Pos_* = 37, *n_NegCont_* = 37, *n_PosCont_* = 37) and 145 undergraduates age 18–35 participated in the AN condition *(n_NegArs_* = 40, *n_Pos_* = 32, *n_NegCont_* = 35, *n_PosCont_* = 38) from the University of Texas at Austin community participated for class credit. Participants were excluded if performance was less than 40% or if 90% or more of their responses were of one category type. The University of Texas at Austin Internal Review Board approved the procedures of this study and written consent was obtained for all participants.

### Materials

#### Emotional Primes

Twenty prime images were presented during training that were taken from 20 yoked images of positive/neutral scenes and 20 yoked images of negative/neutral scenes. Images were a subset of those used in Mather & Nesmith [20] and were matched on arousal and similarity. Matched images were taken from the International Affective Picture System [21] and outside sources on the internet to best match on appearance, complexity, content, and focus of interest while manipulating arousal. Mather and colleagues validated matched images with several raters. Yoked images that were rated as at least 1.5 points apart in arousal on a scale from 1 (low arousal) to 9 (high arousal) and similar to each other with at least a 5.3 rating on a scale of 1 (not at all similar) to 9 (extremely similar) were included.

#### Prototype Stimuli

**Figure 1:**
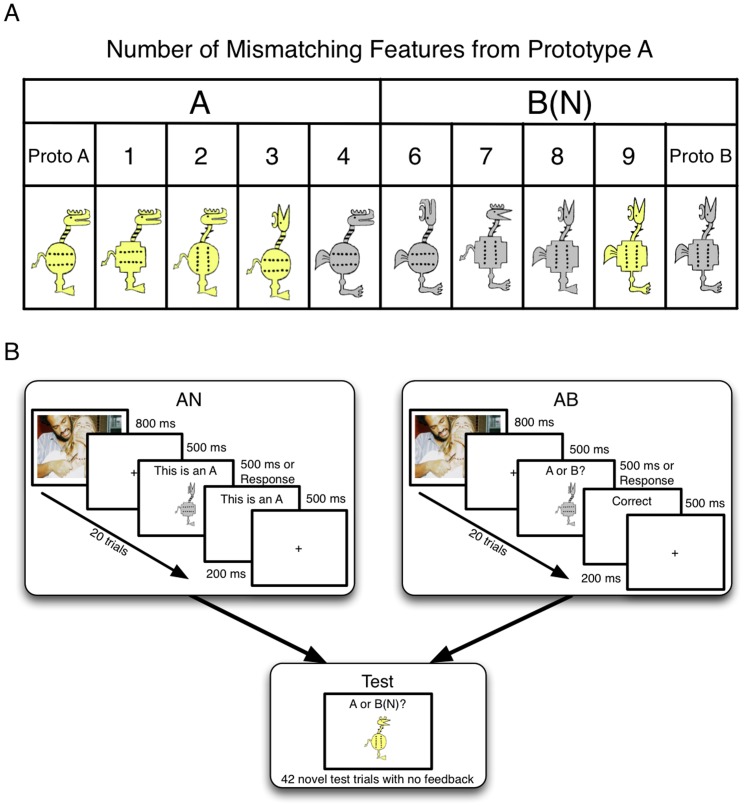
A) Category Structure. B) Participants completed 2 blocks of 20 training trials and 42 test trials (including prototypes and aniprototypes). In this study we presented emotional/neutral emotional prime images 500 ms before stimuli presentation during training to induce a positive or negative mood.

Cartoon animals constructed from 10 binary features such as head orientation (up or forward), body color (grey or yellow), and tail (thin or thick) served as stimuli from a total of 2^10 = ^1024 possible stimuli (Figure 1A). For each participant one stimulus was selected at random to represent the A prototype. The B (or anti) prototype has the opposite value on each feature. Category stimuli were derived by distorting the prototype on one to four randomly selected features. Exemplars that differ from the prototype on five features were not used as they are ambiguous.

### Procedure

We used a 4 emotional prime (negative-arousing, negative-control, positive-arousing, positive-control)×2 task (AB, AN) between-participant design. Training was manipulated between tasks and consisted of 20 trials followed by a test phase with 42 novel stimuli including the prototype and the antiprototype, and equal numbers of A and B items. On each training trial, an emotional or neutral prime was presented for 800 ms, followed by a fixation cross for 500 ms before the stimulus is presented (see Fig. 1B). Each participant completed 2 blocks of 20 training and 42 test trials.

During AB prototype training, participants were shown 10 A and 10 B items in a random order, generated a response and were given corrective feedback. Within each category, 2 training stimuli differed from the category prototype on 1 feature, 3 differed on 2 features, 3 differed on 3 features and 2 differed on 4 features. Across all 10 stimuli within each category, the category typical features were presented 7 or 8 times and the opposite category typical features were presented 2 or 3 times. During AN prototype training, participants were shown 20 A items in a random order with a keystroke required to advance to the next item. Five items differed from the category A prototype on 1, 2, 3, and 4 features.

In both the AB and AN tasks, a 42-trial test phase followed training that included each prototype and 5 stimuli that differed from each prototype on 1, 2, 3 and 4 features. On each test trial, 2 seconds after stimulus onset, the participant was prompted to give an A or B (not A) response with no corrective feedback.

## Results

We were most interested in the effect of emotional primes on stable performance and focused our analysis on the 42-trial final test block (including 40 novel examples and 2 prototypes). We expected and found no performance differences between the positive- and negative-control conditions for the AB task [*M*_NegControl_ = 65, *M*_PosControl_ = 67, *t*(72)<1.0, p = 47] and for the AN task [*M*_NegControl_ = 64, *M*_PosControl_ = 61, *t*(71)<1.0, p = 47]. Therefore, in the following analyses we collapsed across positive and negative control groups within the AB and AN task and refer to them as the “control” group (see Table 1).

**Table 1:** Summary statistics and prototype model parameter estimates for all conditions.

	Emotional Prime	AB	AN
Test Block Accuracy	Negative	0.68 (0.02)	0.58 (0.03)
	Control	0.66 (0.02)	0.62 (0.02)
	Positive	0.59 (0.03)	0.67 (0.02)
Prototype Accuracy	Negative	0.84 (0.04)	0.63 (0.08)
	Control	0.85 (0.04)	0.74 (0.05)
	Positive	0.69 (0.06)	0.88 (0.06)
Antiprototype Accuracy	Negative	0.84 (0.04)	0.48 (0.08)
	Control	0.85 (0.04)	0.64 (0.06)
	Positive	0.69 (0.06)	0.75 (0.08)
Perceptual Discriminability (*c*)	Negative	9.03 (1.71)	2.78 (0.65)
	Control	7.26 (0.99)	4.57 (0.83)
	Positive	4.48 (1.12)	5.44 (1.05)
Max Attention Weight (*w*_max_)	Negative	0.6 (0.04)	0.55 (0.03)
	Control	0.53 (0.02)	0.48 (0.02)
	Positive	0.46 (0.03)	0.46 (0.02)

* Standard Errors are in parentheses.

### Overall Test Accuracy

**Figure 2:**
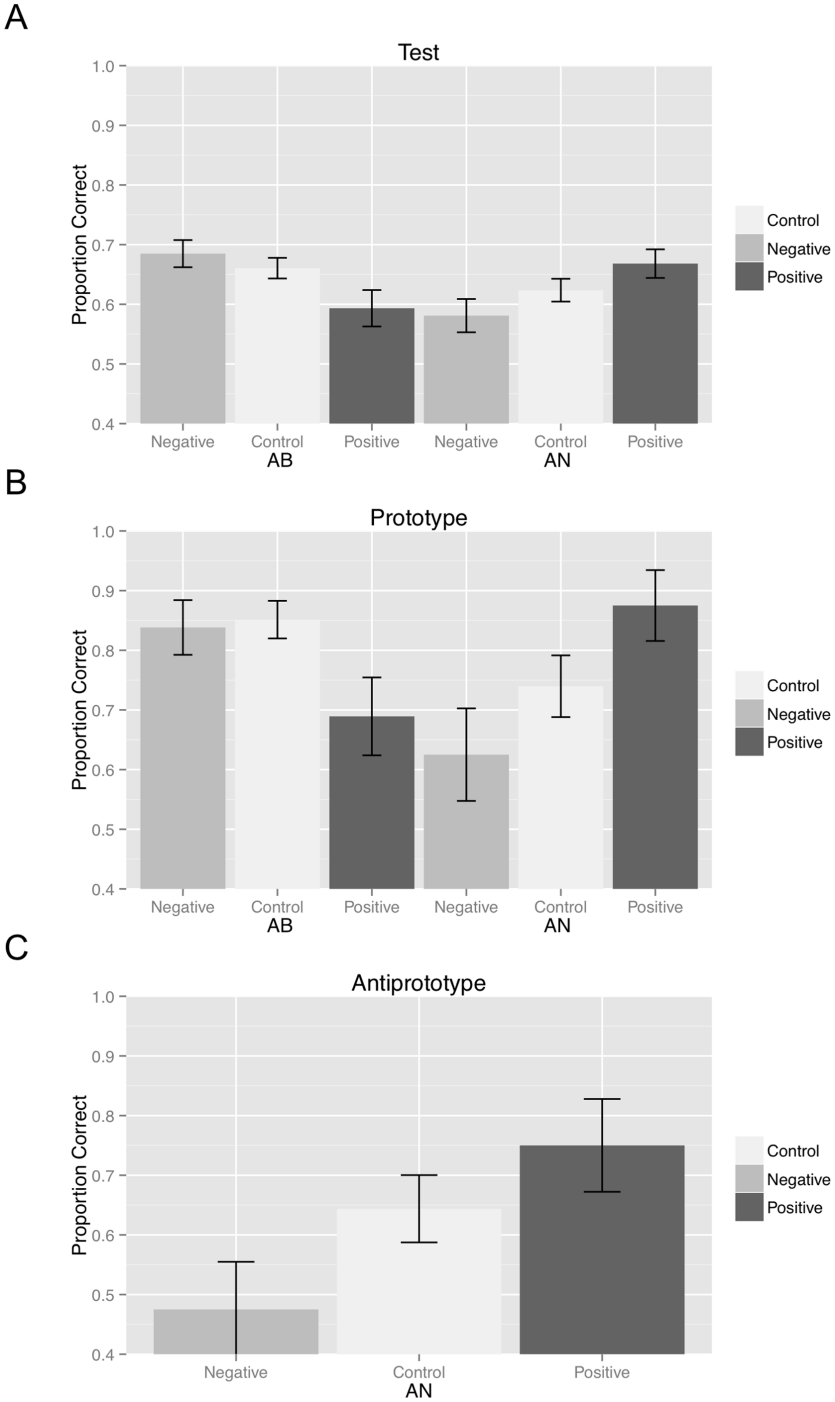
A) Proportion correct for negative, control, and positive emotional prime conditions during test for the AB task and the AN task. B) Proportion prototypes correct for negative, control, and positive emotional prime conditions during test for the AB task and the AN task. C) Proportion antiprototypes correct for negative, control, and positive emotional prime conditions during test for the AN task. Standard error bars included.

Proportion correct was calculated for each participant and a 2 task (AB, AN)×3 emotional prime (negative, control, positive) ANOVA was conducted. There was no main effect of task, F(1, 284) = 2.16, p = 14, *η^2^* = 008, or emotional prime, F(2, 284) = 274, p = 76, *η^2^* = 002. However, there was a significant two-way interaction, *F*(2, 284) = 5.770, *p* = 003, *η^2^* = 04 (Figure 2A).

To decompose the effects of emotional prime valence on AB and AN accuracy, we conducted independent sample t-tests between the negative and positive prime conditions within each task. In the AB task, accuracy was higher in the negative prime condition (*M_Neg_ = 68*) than the positive prime condition (*M_Pos_ = 59*), *t*(69) = 2.37, *p* = 021, *η^2^* = 08. However, in the AN task, accuracy was higher in the positive prime condition (*M_Pos_ = 67*) than the negative prime condition (*M_Neg_ = 58*), *t*(70) = 2.30, *p* = 024, *η^2^* = 07. This indicates that task performance depends on the valence of emotional primes with negative primes enhancing AB task performance and positive primes enhancing AN task performance. Performance in the control condition fell between the positive and negative conditions for both tasks and there was no statistical difference, *t*(145) = 1.43, *p* = 154, *η^2^* = 014, *ns*, between the control prime conditions in the AB and AN task (*M_AB_ = *66, *M_AN_ = *62) suggesting that the tasks are of equivalent difficulty.

### Prototype Accuracy

Prototype accuracy was calculated for each participant. In the AB condition the average accuracy for the A and B prototypes was calculated and in the AN condition accuracy for the A prototype was calculated. A 2 task (AB, AN)×3 emotional prime (negative, control, positive) ANOVA was conducted on prototype accuracy. There was no main effect of task, *F*(1, 284) = 2.27, *p* = 13, *η^2 = ^*008, or emotional prime, *F*(2, 284) = 84, *p* = 43, *η^2 = ^*006. However, there was a significant two-way interaction, *F*(2, 284) = 5.503, *p* = 005, *η^2 = ^*04 (Figure 2B). Post-hoc analyses suggested that prototype accuracy was marginally higher in the negative prime condition (*M_Neg_ = 84*) than the positive prime condition (*M_Pos_ = 69*), *t*(69) = 1.84, *p* = 07, for AB learning, but was higher in the positive prime condition (*M_Pos_ = 87*) than the negative prime condition (*M_Neg_ = 62*), *t*(70) = 2.46, *p* = 016, for AN learning. This indicates that prototype accuracy in each task depends on the valence of emotional primes.

### Antiprototype Accuracy

Antiprototype accuracy was examined in the AN task only since both prototypes were trained in the AB condition. We conducted a one-way ANOVA examining priming condition (positive, control, negative) within the AN task. There was a main effect of prime, *F*(2, 142) = 3.09, *p* = 05, *η^2 = ^*04, T-tests between the negative and positive prime conditions indicated that accuracy was higher in the positive prime condition (*M_Pos_ = 75*) than the negative prime condition (*M_Neg_ = 48*), *t*(70) = 2.43, *p* = 018, *η^2^* = 08. Together, these results indicate that prototypes and antiprototype accuracy depends on the valence of emotional primes and training where negative primes improve accuracy in the AB task and positive primes improve accuracy in the AN task.

### Computational Models

The accuracy based analyses support our prediction that positive emotionally-valenced arousal will accentuate perception-mediated AN learning, whereas negative emotionally-valenced arousal will accentuate memory-mediated AB learning. However, these analyses provide no information regarding our prediction that the locus of these effects is in emotional arousal's influence on attentional scope. Specifically, we hypothesized that AN learning requires broad attentional focus and thus will be enhanced by positive emotional arousal, whereas AB learning requires narrow attentional focus and thus will be enhanced by negative emotional arousal. To test these predictions we turn to computational modeling techniques. We applied simple prototype models to each individual's data [22]–[24].

The model assumes that on each trial, the participant calculates the attention-weighted Euclidean distance between the current stimulus (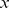) and the prototype for the categories (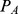 for category A, and 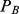 for category B(N)). The attention weights stretch and shrink the perceptual space along each stimulus dimension with larger attention weights stretching the space (increasing dimension-level discriminability). The (city block) distance between 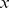 and 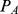 is calculated as:



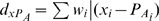
(1.1)where 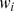 represents the attention-weight associated with dimension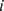. The attention weights are constrained to sum to 1, yielding 9 free 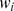 parameters. The stimulus' binary value for dimension 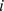 is denoted by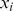, and prototype A's binary value for dimension 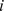 is denoted by 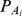. 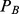 is calculated on each trial using the same method. The predicted probability of responding A to a stimulus, 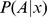, is calculated as:



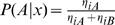
(1.2)where 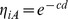 (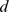
is calculated in Eq. 1.1). The 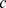 parameter represents the perceptual sensitivity of the system, and is the 10th free parameter. Larger values of 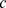 stretch the perceptual space uniformly leading to greater overall discriminabilty across stimuli. For each participant, we fit the model to 42 test items from the test block using maximum likelihood procedures.

### Attentional Focus

We predict that positive emotional primes broaden attention whereas negative emotional primes narrow attention. As a test of this hypothesis we identified the dimension with the maximum attention weight (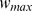). A large 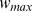 implies narrow attention and a small 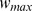 implies broad attention. We conducted a 2 task (AB, AN)×3 emotional prime (negative, control, positive) ANOVA on the 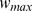 values and found no main effect of task and no interaction. As predicted, there was a main effect of emotional prime, *F*(2, 284) = 7.036, *p = *001, *η^2^* = 047, where negative primes were associated with larger 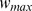 (*M_Neg_ = *57) and positive primes were associated with smaller 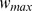
(*M_Pos_ = *46) values. This indicates that negative emotional primes narrow attention while positive primes broaden attention.

In addition to looking at maximal attentional weight, we examined attentional scope by calculating the number of dimensions needed to capture ninety-five percent of the attentional weights (..). Here smaller values indicate that fewer dimensions are attended to and thus attention is narrow. We conducted a 2 task (AB, AN)×3 emotional prime (negative, control, positive) ANOVA on the number of dimensions needed to capture 95% of attentional weights and found no main effect of task and no interaction. However, as seen with maximal attentional weights, there was a main effect of emotional prime, *F*(2, 284) = 3.93, *p = *02, *η^2^* = 03, where negative primes were associated with smaller 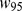 (*M_Neg_ = *3.3) and positive primes were associated with larger 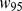 values (*M_Pos_ = *3.7). This indicates the effects of emotion on attentional scope are robust to multiple measures of attention.

### Perceptual Discriminability

**Figure 3:**
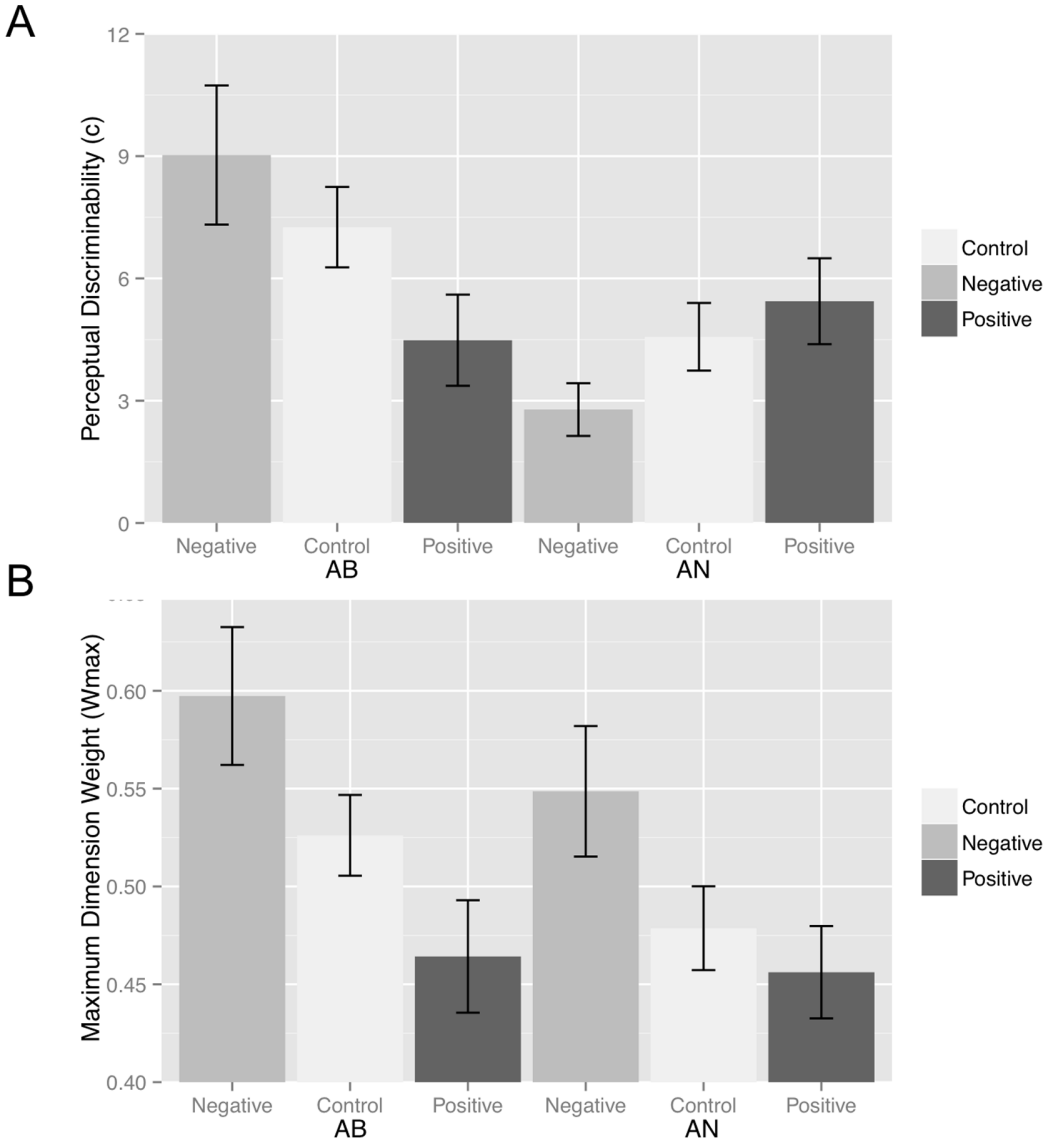
*A*) Perceptual discriminability parameter estimates (*c*) X emotional primes (negative, positive, neutral) for the AB task and the AN task. *B)* Maximum dimension weight parameter (*w*_max_) estimates X emotional primes (negative, positive, neutral) for the AB task and the AN task. Standard error bars included.

We also predict that narrow attention facilitates AB learning whereas broad attention facilitates AN learning thus leads to increased perceptual discriminability. As a test of this hypothesis we conducted a 2 task (AB, AN) X 3 emotional prime (negative, control, positive) ANOVA on the perceptual discriminability (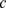) values (Figure 3A) and found a main effect of task, F(1, 284) = 8.299, p = 004, *η^2 = ^*028, no main effect of emotional prime, *F*(2, 284) = 429, *p* = 65, *η^2^* = 003, and an interaction *F*(2, 284) = 6.628, *p* = 002, *η^2 = ^*05. In support of our prediction, post hoc analyses revealed that perceptual discriminability was higher in the negative prime condition (*M_Neg_ = 9.03*) than the positive prime condition (*M_Pos_ = 4.48*), *t*(69) = 2.26, *p* = 027, *η^2^* = 07 for the AB task, but was higher in the positive prime condition (*M_Pos_ = 2.78*) than the negative prime condition (*M_Neg_ = 5.44*), *t*(70) = 2.24, *p* = 028, *η^2^* = 07 for the AN task.

### Parameter Estimates and Performance

To further test our assumption that narrow attention facilitates AB learning and broad attention facilitates AN learning, we correlated AB and AN accuracy with the maximum attentional weight (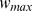) and perceptual discriminability (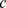) parameter values. A larger maximum attentional weight (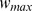) was associated with increased performance in the AB task, *r^2^ = *23, *F*(1,143) = 8.03, *p = *005, but not the AN task, *r^2^ = *−06, *F*(1,143) = 045, *p*  = 50, *ns.* In addition, increased perceptual discriminability (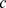) was associated with increased performance in both the AB task, *r^2^ = *63, *F*(1,143) = 95.50, *p<*001, and the AN task, *r^2^ = *48, *F*(1,143) = 41.92, *p*<.001 (Figure 3B). Thus, focused attention was associated with improved accuracy in the AB task only, whereas greater perceptual discriminability was associated with improved accuracy both the AB and AN tasks.

## Discussion

The effects of arousal on attention during perception and memory have been well studied, but little work has examined the effects of arousal on learning. The current study represents the first to examine arousal's effects on learning in dissociable perceptual-representation-mediated and declarative-memory-mediated learning. The *Arousal-Biased Competition* theory (ABC) states that arousal exaggerates ongoing competitive attentional processes between high- and low-priority stimuli [11], [7]. High-priority items are given more attentional resources at the expense of low-priority items, but no strong claims are made regarding the effects of valence on attention. Fredrickson and colleagues have demonstrated that positively-valenced arousal broadens the scope of attention whereas negatively-valenced attention narrows the scope of attention [14], [16]. However, Gable and Harmon-Jones argue that high approach-motived positive arousal narrows attention as seen under negative arousal [25], [26]. Thus, while attention for high-priority items is enhanced, it is unclear whether scope of this attention is valence-dependent.

The overriding aim of this study was to determine how positive and negative emotionally arousing primes affect attentional resources and performance in two prototype learning systems – one mediated by declarative memory and rule-based processing and another mediated by a strong perceptual representation. Prior research suggests that negative emotional primes narrow attention, which should facilitate targeting features for verbalizable rules, while positive emotional primes broaden attention, which should facilitate a strong perceptual representation. Though the effects of priming on cognition are subtle, which is often reflected in modest effect sizes (see [27], the predicted interaction between valence and system is an important one. Prototype learning is an ideal paradigm to test this hypothesis because dissociable declarative memory and perceptual representation systems have been demonstrated [5] and computational models are available that provide estimates of attentional scope.

### Performance

We hypothesized that the valence of emotional primes (positive, negative) affects attentional scope during training and therefore interacts with the system that mediates learning in each task (AB, AN). The results supported our predictions. In the AB task, negative emotional primes improved overall and prototype accuracy relative to positive emotional primes, whereas in the AN task positive emotional primes improved overall, prototype, and antiprototype accuracy relative to negative emotional primes.

### Attention

Mather and colleagues suggest that priority items seen after high-arousal primes benefit from enhanced attentional resources regardless of valence in their theory of *Arousal Biased Competition* [11]. Our computational modeling data indicates that this relationship is more complex and the valence of emotional primes affects the scope of enhanced attention. In the negative emotional prime condition, greater attentional weight was placed on one stimulus dimension compared to the positive emotional prime condition regardless of task suggesting that negative emotional primes narrow and positive emotional primes broaden attentional scope for subsequent stimuli.

### Perceptual Sensitivity

Perceptual sensitivity (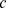) depends on both the valence of the emotional prime (negative, positive) and the task (AB, AN) and tracks performance. In the AB task those in the negative emotional prime condition were more sensitive to perceptual differences between exemplars than those in the positive emotional prime condition. There is a significant reversal of this pattern in the AN task. This indicates that global perceptual sensitivity aids in both the perceptual representations that aid familiarity judgments during the AN task and declarative memory processes that aid in verbalizable rule formation in the AB task.

### Stimulus Dimensionality

The present research examined AB and AN prototype category learning using cartoon stimuli composed of 10 binary value features. Previous research examining these learning systems has looked at the categorization of high-dimensional stimuli such as random Posner dot patterns [28], [24], [29] or low-dimensional stimuli such as Gabors [30], [31], [32]. One question that this raises is whether the current findings would generalize to these other types of stimuli. Much of the work utilizing dot patterns examined AN category learning. Reber et al. (1998) looked at functional activation during test after participants were trained on distortions from one Posner dot pattern prototype. This task is analogous to our AN task as both examine stimuli with high number of features (patterns of 9 dots in the Posner task) that are distorted from one prototype. Both Reber et al (1998) and Zeithamova et al (2008) found changes in activation in the posterior occipital cortex suggesting an overlapping neural network. Thus, we predict that the effects of emotional primes on AN learning would generalize to performance during the Posner dot pattern task.

Predictions for Gabor patch stimuli are less clear. In fact, the results from one study using Gabor patch stimuli appear counter to what we observed. Nadler et al (2012) found that induced positive mood improved learning in a rule-based task where participants categorized Gabor patches by one dimension (frequency). Improved performance is attributed to increased prefrontal dopamine enhancements to cognitive flexibility, which aids in rule development (see [33]). Though positive affect helps rule acquisition in this case, it is likely that the relative importance of cognitive flexibility and attentional scope differ as a function of the dimensionality of the stimuli. High dimensional stimuli require narrow attentional scope in order to select salient features during rule development. Gabor patches are simple stimuli that only vary on two dimensions (frequency and orientation) and attentional scope is not as important in determining learning outcomes. Thus, it is possible that stimulus dimensionality is an important factor that interacts with learning system and mood in determining learning outcomes. Future work should better address these conflicting findings by manipulating the number of stimulus feature dimensions and comparing emotionally valenced priming effects directly with mood induction effects.

## Conclusions

These data suggest that the valence of task-irrelevant emotional arousal is a critical factor in determining learning outcomes. Negative emotional primes narrow attentional scope optimizing memory-mediated learning that depends on concrete verbalizable rules. Positive emotional primes broaden attentional scope optimizing perception-mediated learning that depends on global perceptual representations of stimuli.
